# Laparoscopic management of foramen of Winslow incarcerated hernia

**DOI:** 10.1186/s40792-016-0139-4

**Published:** 2016-02-06

**Authors:** Ronald Daher, Laura Montana, Jarrah Abdullah, Antonio d’Alessandro, Elie Chouillard

**Affiliations:** Department of General and Minimally Invasive Surgery, Centre Hospitalier Intercommunal Poissy/Saint-Germain-En-Laye, 10, rue du Champ Gaillard, 78300 Poissy, France

**Keywords:** Winslow, Laparoscopy, Puncture, Incarcerated hernia, Caecopexy

## Abstract

Foramen of Winslow hernia (FWH) is a rare and often overlooked diagnosis with a high mortality rate. Widespread availability of cross-sectional imaging allows early diagnosis and prompt management. In this setting, before ischemia occurs, explorative laparoscopy would be the most suitable approach. Experience, however, remains sparse, and technical difficulties may be encountered. This is the case of a 38-year-old Caucasian woman who presented to the emergency department for a sudden epigastric pain. Physical exam was unremarkable, and routine blood tests were within normal range. An abdominal computed tomography (CT) scan confirmed the diagnosis of ileocaecal herniation through the foramen of Winslow. Under urgent laparoscopy, the caecum appeared viable but incarcerated in the lesser sac. Caecal puncture was the key to achieving atraumatic reduction of the hernia and bowel salvage.

## Background

Since the first description by Blandin in 1834, foramen of Winslow hernias (FWH) has been estimated to represent 8 % of all internal hernias [1, 2]. Different types of viscera can be involved (small bowel, caecum, transverse colon, gallbladder) with epigastric pain being the most common symptom [3]. Different factors are known to predispose to the FWH, including different anatomical abnormalities, such as enlarged foramen of Winslow, redundancy of mesentery/mesocolon, incomplete adhesion of the bowel to the posterior abdominal wall or acquired factors that increase intra-abdominal pressure such as pregnancy or postprandial periods [1–4]. Because of non-specific signs and symptoms [2, 3], the diagnosis is often delayed with a historical mortality rate approaching 50 % [4]. This rate was dramatically reduced to 5 % in most recent series, consequently to widespread usage of cross-sectional imaging with computed tomography (CT) [5].

The treatment of FWHs remains, however, challenging. The vast majority of cases are managed with a laparotomy [2, 6, 7], and only a few recent reports favour the laparoscopic approach [1, 8–13]. When the involved bowel remains viable, the treatment comes down to hernia reduction and prevention of recurrence. Laparoscopic experience, however, remains sparse and technical difficulties may be encountered such as reduction of the incarcerated bowel through the non-expandable foramen of Winslow.

We report here the case of a 38-year-old woman presenting with an ileocaecal FWH, successfully treated under laparoscopy. We will especially focus on the laparoscopic technique of caecal puncture, allowing atraumatic reduction of the hernia as well as sparing of the incarcerated bowel.

## Case presentation

A 38-year-old woman presented to the emergency department with a sudden onset of epigastric pain for 12-h duration. Pain was associated with nausea without vomiting or changes in bowel habit. The pain was unresponsive to auto medications (paracetamol or proton pump inhibitors). There was no history of other drug intake or alcohol abuse. The patient’s past surgical history was unremarkable except for caesarean section that followed a difficult vaginal delivery, 2 years prior to presentation.

Physical examination revealed an apyretic hemodynamically stable patient. She was found to have epigastric tenderness on palpation but no guarding or signs of peritoneal irritation. Bowel sounds were present. The remaining physical examination was otherwise unremarkable.

Blood tests were within normal limits including no leucocytosis, negative C-reactive protein and normal liver and pancreatic enzymes.

**Fig. 1:**
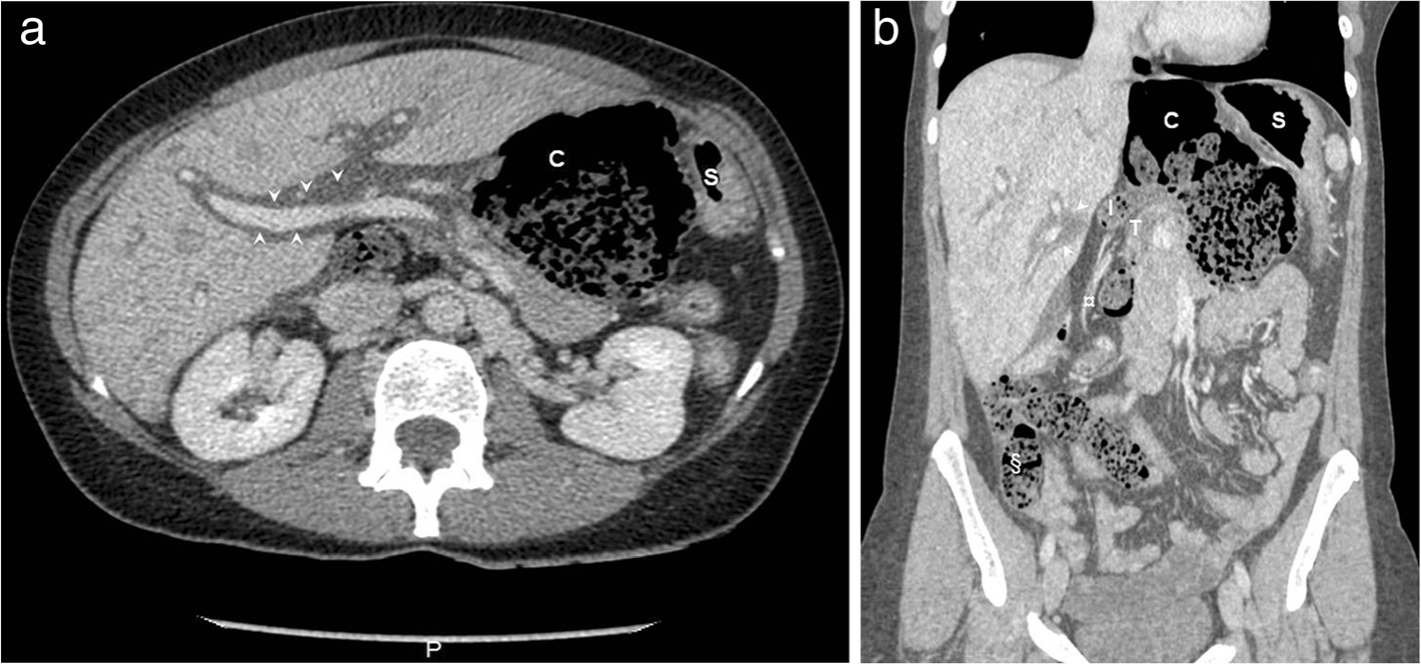
Abdominal contrast-enhanced computed tomography. **a** Axial section shows caecal herniation (*C*) in the lesser sac causing anterior and lateral displacement of the stomach (*S*). Edematous infiltration seen along the portal pedicles (*arrow heads*). **b** Coronal reconstruction demonstrates the ileocaecal pedicle (*¤*), the terminal ileum (*I*) and the transverse colon (*T*) trapped within the foramen of Winslow. Ileal loops in the *lower right quadrant* present with the “faeces sign’ (*§*) in the absence of the caecum

An abdominal intravenous contrast CT scan demonstrated a herniated caecum through the foramen of Winslow (measured at 3.5-cm of diameter), representing a 7.5-cm faeces-containing collection in the lesser sac displacing the stomach anteriorly and to the left. There was an associated ‘faeces sign’ in the small bowel loops (Fig. 1).

The patient underwent an urgent exploratory laparoscopy using a 12-mm supra-umbilical port and three 5-mm working ports in the left lumbar, right lumbar and epigastric regions. The caecum and terminal ileum along with the appendix had herniated through the foramen of Winslow. There was free serous intraperitoneal fluid. After division of the gastrohepatic ligament, the caecum was found to be markedly distended but undoubtedly well-vascularized without lacerations. Edematous infiltration was found within the porta hepatis.

**Fig. 2:**
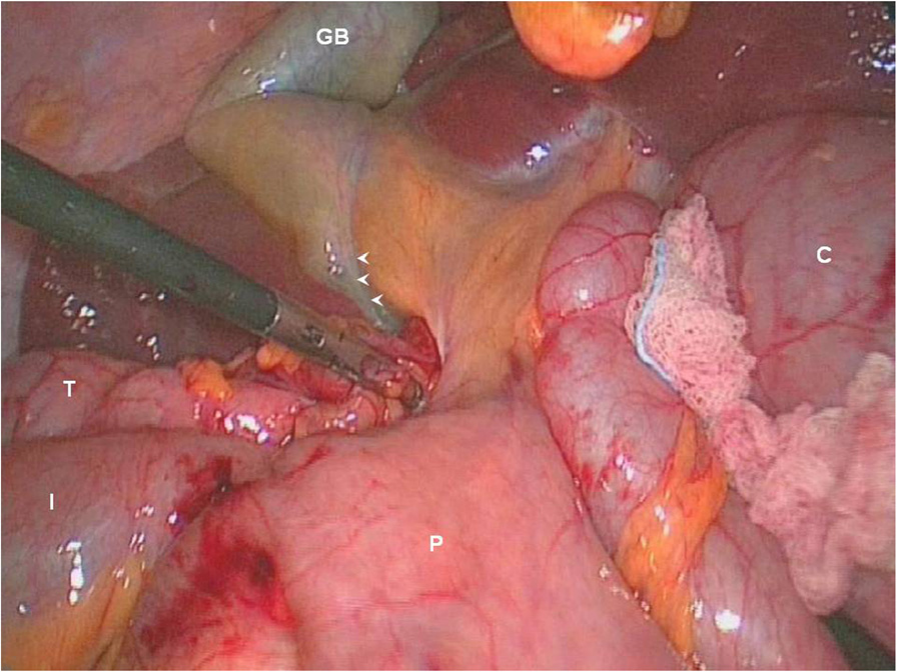
Attempts of hernia reduction. Intraoperative view shows incarcerated but viable caecum (*C*). Concomitant push-pull manoeuver with right and left instruments failed to reduce the herniated bowel. Transverse colon (*T*), terminal ileum (*I*), pylorus (*P*), gallbladder (*GB*), edematous infiltration along the porta hepatis (*arrow heads*)

**Fig. 3:**
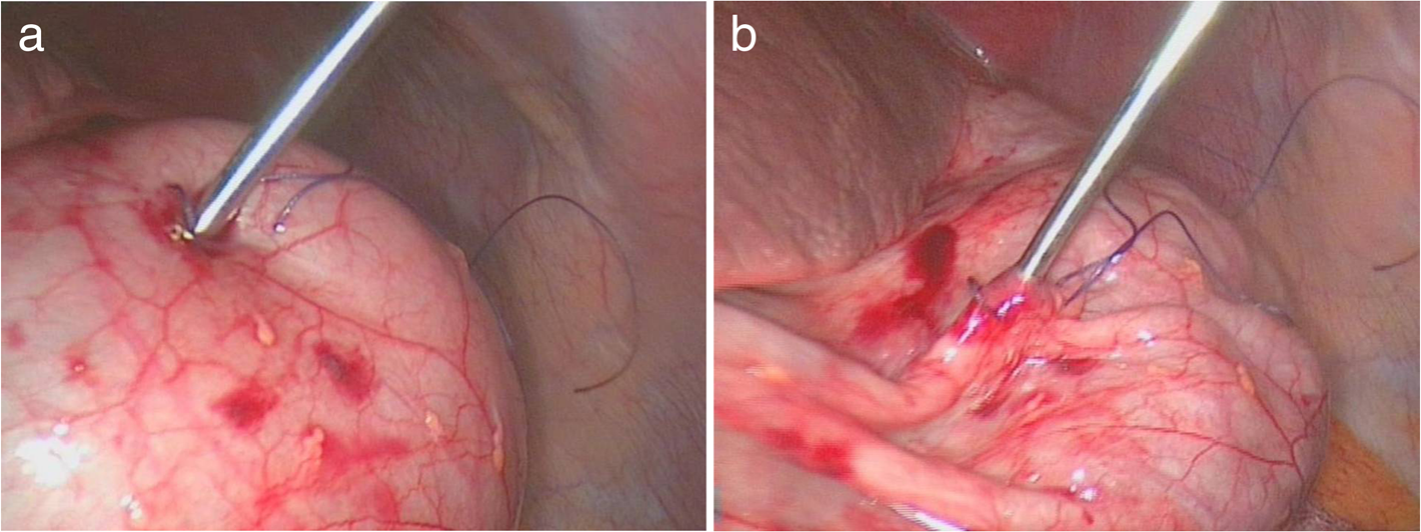
Caecal decompression. **a** Caecal puncture using a Veress needle. **b** Deflated bowel allowing atraumatic reduction of the hernia

Several attempts for hernia reduction have been executed, using one hand for proximal traction of the colon, appendix and even the dilated ileum and the other for applying opposing pressure using a gauze-protected forceps (Fig. 2). These manoeuvres had to be stopped because of a minimal tear to the mesoappendix. Thereafter, puncture site on the caecum was prepared with a purse string using Coated VICRYL® 000 (polyglactin 910, Sutures, ETHICON). Decompression of the caecum was performed using a Veress needle introduced through the abdominal wall (Fig. 3). Thereafter, the purse string was tight concomitantly with the removal of the needle by the assistant. No leakage was noticed, and consequently no lavage/drainage was performed. Subsequent reduction of the hernia was straightforward, and careful inspection ruled out organ injury. No colonic elongation was noticed; instead, a lack of posterior wall attachment of the ascending colon was present, justifying a caecopexy with a non-absorbable suture along and a prophylactic appendicectomy.

The postoperative course was uneventful. Full oral intake was tolerated on postoperative day 1, and the patient was discharged on day 2. At the 6-month follow-up, the patient did not show any suggestive signs or symptoms of complications or recurrence.

### Discussion

Among the 200 reported cases of FWH, the laparoscopic approach has been rarely used and solely in the most recent papers [1, 8–14]. Among them, hernia reduction was inconstantly associated with caecopexy when the ileocecal portion was involved [8–10, 13]. Right colectomy is indicated for bowel ischemia or systematically achieved by some authors [15]. Needle bowel decompression has been previously described for reduction of incarcerated hernias but only performed under laparotomy and/or followed by right colectomy [1, 15, 16]. This is the first case to report laparoscopic puncture emptying of an otherwise healthy but incarcerated colon in order to achieve hernia reduction and avoid bowel resection.

Historically, FWHs have been impinged by a significant mortality rate reported as high as 50 % [4]. This was the consequence of delayed diagnosis in the setting of a rare disease lacking specific symptoms or peritoneal signs (due to strangulation located in the lesser sac). Abdominal CT scan is presently accepted as the imaging modality of choice allowing preoperative diagnosis of FWHs [2, 3, 7, 11]. Suggestive CT findings such as air-fluid level in the lesser sac which taper toward the foramen, presence of mesentery between the portal vein and inferior vena cava, absence of ascending colon in the right gutter and anterolateral displacement of the stomach have been recognized in our case and allowed timely surgical management.

It is now established that urgent abdominal exploration significantly reduces morbidity and mortality rates when performed before irreversible ischemia occurs [2, 11]. Laparotomy has traditionally been advocated as the preferred approach [2, 6, 7, 15]. Some recent reports, however, showed that laparoscopy offers exploratory possibilities, treatment options and enhanced recovery in this particular setting of prompt management [1, 8, 10, 11, 14]. For incarcerated but viable hernias, the Kocher manoeuvre has been proposed to enlarge the foramen of Winslow [17]. This technique, however, is potentially difficult to realize due to distorted anatomy and inflammatory adhesions.

Alternatively, we advocate puncture decompression of the incarcerated bowel as a feasible and safe technique. We consider that it is preferable to suture a planned puncture before bowel reduction than to repair an irregular tear that would have occurred during excessive trauma. An uncontrolled tear in a weakened hollow organ exposes the risk of faecal contamination and could potentially necessitate organ resection. Planned perforation of a hollow viscus can be safely accomplished under laparoscopy without compromising the postoperative course. In this case, bowel movements recovered soon after surgery allowing full oral intake and discharge on postoperative day 2.

Treatment of viable non-ischemic FWHs is not standardized [7]. Some authors limit the procedure to hernia reduction, especially when achieved under laparoscopy [8–10, 13]. Although recommended by some authors [2, 18], there are no current guidelines for foramen of Winslow closure [1]. This exposes to the serious risk of injury of the porta hepatis while the inflammatory postoperative adhesions seem to most often close the foramen [3]. Alternatively, the foramen of Winslow can be packed with omentum [11, 14]. Systematic right colectomy, even for viable bowel, has been proposed when appropriate peritonization cannot be achieved due to redundant colon [19–21]. We agree that lack of posterior attachment of the caecum and ascending colon is a confirmed risk factor for FWH and consider that caecopexy to the lateral wall is the most suitable option when the bowel is deemed viable after reduction [2, 6]. Being easily achieved under laparoscopy, it combines the advantages of avoiding porta hepatis injury, protecting against theoretical recurrence and allowing bowel sparing.

## Conclusions

In conclusion, laparoscopy appears as the ideal surgical approach for early managed foramen of Winslow hernias. In the case of viable but incarcerated bowel, puncture decompression is a feasible and safe technique offering atraumatic hernia reduction and avoidance of bowel resection.

## Consent

Written informed consent was obtained from the patient for publication of this case report and any accompanying images. A copy of the written consent is available for review by the Editor-in-Chief of this journal.

## Abbreviations

CT: computed tomography
FWH: foramen of Winslow Hernia
